# OpenML: Insights from 10 years and more than a thousand papers

**DOI:** 10.1016/j.patter.2025.101317

**Published:** 2025-07-03

**Authors:** Bernd Bischl, Giuseppe Casalicchio, Taniya Das, Matthias Feurer, Sebastian Fischer, Pieter Gijsbers, Subhaditya Mukherjee, Andreas C. Müller, László Németh, Luis Oala, Lennart Purucker, Sahithya Ravi, Jan N. van Rijn, Prabhant Singh, Joaquin Vanschoren, Jos van der Velde, Marcel Wever

**Affiliations:** 1 Department of Statistics, LMU Munich, Munich, Germany; 2 Munich Center for Machine Learning (MCML), Munich, Germany; 3 Department of Mathematics and Computer Science, Eindhoven University of Technology, Eindhoven, the Netherlands; 4 Microsoft, Mountain View, CA, USA; 5 Weierstrass Institute for Applied Analysis and Stochastics, Berlin, Germany; 6 Max Planck Institute for Demographic Research, Rostock, Germany; 7 Dotphoton, Zug, Switzerland; 8 Department of Computer Science, University of Freiburg, Freiburg, Germany; 9 Department of Computer Science, University of British Columbia, Vancouver, BC, Canada; 10 Leiden Institute of Advanced Computer Science, Leiden University, Leiden, the Netherlands; 11 L3S Research Center, Leibniz University Hannover, Hannover, Germany

**Keywords:** machine learning, artificial intelligence, open science, networked science, benchmarking, FAIR data, reproducibility, meta-learning, automated machine learning, OpenML

## Abstract

OpenML is an open-source platform that democratizes machine-learning evaluation by enabling anyone to share datasets in uniform standards, define precise machine-learning tasks, and automatically share detailed workflows and model evaluations. More than just a platform, OpenML fosters a collaborative ecosystem where scientists create new tools, launch initiatives, and establish standards to advance machine learning. Over the past decade, OpenML has inspired over 1,500 publications across diverse fields, from scientists releasing new datasets and benchmarking new models to educators teaching reproducible science. Looking back, we detail and describe the platform’s impact by looking at usage and citations. We share lessons from a decade of building, maintaining, and expanding OpenML, highlighting how rich metadata, collaborative benchmarking, and open interfaces have enhanced research and interoperability. Looking ahead, we cover ongoing efforts to expand OpenML’s capabilities and integrate with other platforms, informing a broader vision for open-science infrastructure for machine learning.

## Highlights

• Over the past decade, OpenML has democratized and facilitated machine learning
• The OpenML ecosystem impacted over 1,500 studies across various research areas
• Building and maintaining OpenML is a challenging, continuous community effort
• Our experiences help guide the future of open-science machine-learning infrastructure

## Teaser

The authors reflect on 10 years of OpenML, the open-source platform that turns machine-learning experiments into open, linked, and reusable knowledge. They present the current state of the OpenML ecosystem and its design and assess how community-curated datasets, tasks, and benchmark suites have powered more than 1,500 studies, advanced machine-learning research, and improved reproducibility. Lastly, they share lessons from a decade of building, maintaining, and expanding OpenML to guide the future of open-science infrastructure for machine learning.

## Introduction

Machine learning (ML) is transforming scientific research, yet its trustworthiness and progress hinge on reproducibility and transparency in data provenance, model design, training, and evaluation, which remain elusive despite wide-ranging efforts.^1^^,^^2^^,^^3^^,^^4^

Imagine a world where all ML research can be easily shared in full transparency online, where richly described datasets are interconnected with all open-source code that uses them, the resulting trained models, reproducible benchmarks thereof, and all papers and other documents with interesting findings. Imagine that all of these artifacts are described and hosted in standardized ways that make them interoperable and reusable across platforms and tools. This would allow scientists across disciplines to easily find the best ML techniques, understand their capabilities, and reuse them in new settings, building effortlessly on each other’s work.

The wider open-science movement has made great strides toward this vision, advocating for open access to publications, data, code, and results, creating guidelines and tools to describe them (e.g., Findable, Accessible, Interoperable, Reusable [FAIR] data^5^ and Jupyter Notebooks^6^) and platforms to host them (e.g., arXiv, GitHub). However, reproducing and reusing ML research, in particular, remains challenging due to the complexity and heterogeneity of datasets, models, and computational environments, requiring much more detailed standards. Moreover, interdependent objects (e.g., data, code, and benchmarks) are still fragmented across specialized platforms, and, as the underlying technologies evolve, once-valuable resources can quickly become unusable or incompatible, a phenomenon known as technology drift.

In response to these challenges, we created OpenML,^7^ a fully open-source platform that fosters collaboration by enabling users to upload and share datasets in uniform standards, define precise ML tasks to work toward common goals, and conveniently share ML workflows and model evaluations directly from ML tools all on the same platform. OpenML provides a rich, uniform metadata structure to capture all the information in the ML life cycle and facilitate long-term reuse, as well as benchmarking suites to run more systematic model evaluations.

Over the past decade, OpenML has evolved into a vibrant, community-driven resource, inspiring over 1,500 publications while supporting research across diverse fields. With scientists releasing new datasets, developers benchmarking new algorithms, and educators teaching data science, OpenML has become a cornerstone for enhancing data-repository practices.

In this paper, we reflect on more than a decade of building, maintaining, and expanding OpenML. We examine how its design, centered on rich metadata, collaborative benchmarking, and open interfaces, has enabled large-scale comparisons of ML algorithms.^8^ Specifically, we perform a quantitative analysis across all research papers (until 2024) that have referenced OpenML papers to understand its adoption across communities. Beyond this analysis, we highlight several communities that have specifically adopted OpenML, including the reproducible benchmark community, the AutoML community, as well as the education community. We also showcase community-driven successes, including major reproducibility initiatives, and discuss ongoing efforts to expand the platform’s capabilities. Our overarching goal is to show how OpenML’s journey can inform the broader vision for open-science infrastructure in ML.

We start by explaining how OpenML was designed and developed, and then we position OpenML in the context of related work. Next, we analyze the impact that OpenML has had on ML research. Finally, we reflect on lessons learned and what opportunities remain to build a truly networked science environment for open ML.

## Building OpenML

OpenML was designed to allow easy sharing and access to unified, structured ML experiment data. We achieve this by decomposing ML experiments into modular components and making the data available through the OpenML platform. Beyond technical aspects, we also discuss community, funding, and challenges in this section, in particular for reproducibility. We defer a discussion of ways in which we try to collaborate beyond OpenML by breaking the silos of data repositories to the end of the related work section.

### Decomposing ML experiments

A prototypical ML experiment consists of training a model with an ML algorithm on training data, producing predictions on test data, and evaluating those predictions. This is often repeated under resampling and across multiple algorithms and datasets in benchmark experiments. On OpenML, the prototypical ML experiment is broken down into distinct concepts: datasets, tasks, flows, runs, and collections.

**Figure 1:**
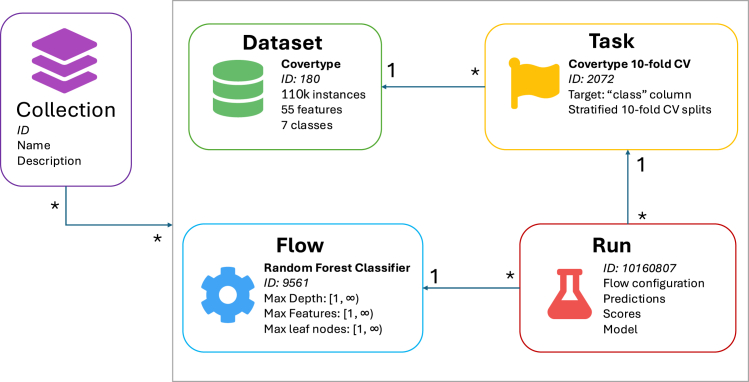
The relationships between the concepts OpenML uses to define an ML experiment The annotation of the connections denotes the cardinality; e.g., a task typically refers to exactly one dataset, but one dataset may be referenced by many tasks. A collection is a set of entities of the same type. We currently only support collections of tasks, also called benchmarking suites, and collections of runs, which are called studies.

Figure 1 illustrates the relationship between these concepts. Datasets are a core concept of ML but do not constitute a scientific task. Tasks describe for each ML experiment which data should be used and what the evaluation procedure is, and they define the train-test splits. When an algorithm (flow) is then trained and evaluated, the results (e.g., predictions) are captured in a run. Anyone can upload any part of their ML experiment, which makes these objects freely available to other users. They are provided through various application programming interfaces (APIs), and each concept has its own standardized metadata. This makes it easy for people to build on each other’s work, for example, for a community to accept a standardized benchmarking suite, or to study the effect of algorithm hyperparameter configurations by inspecting uploaded run results.

#### Datasets

The vast majority of datasets on OpenML are structured, tabular datasets. These datasets span many different domains, such as healthcare, finance, and computer science, and consist of data organized into rows and columns. More recently, OpenML has added support for other modalities whose datasets can be defined by a header table combined with a file structure, supporting task types from the domain of, e.g., computer vision. One prominent example of image data on OpenML is Meta-Album,^9^ a large collection of datasets with images from various domains to facilitate, among other things, transfer learning, meta-learning, and continual learning research.

Each OpenML dataset is described by metadata that include the name, description, author, license, and other information about the dataset. It is also possible to provide crucial contextual information by annotating individual features (columns) in the dataset. Additionally, meta-features that describe the data values are also automatically computed and available. This includes simple meta-features such as the number of rows and columns in tabular data but also more complex types, including statistical meta-features (e.g., the mean standard deviation of all numerical columns), information-theoretic meta-features (e.g., the mean information gain of all categorical attributes), and landmarkers^10^ (e.g., the predictive performance of a simple classifier trained on the data). The metadata are available in a standardized way, which makes it easy to programmatically sort through the thousands of datasets on OpenML and select the datasets that fit certain criteria or use it for meta-learning.^11^

#### Tasks

A dataset in itself does not constitute a scientific task. There are many ways in which an ML model can be evaluated on any given dataset, and this needs to be properly specified to make evaluations reproducible and to clearly communicate to other researchers how models should be meaningfully evaluated.

In OpenML, all these aspects are described in a task object, and any dataset may have many different tasks associated with it. An OpenML task always has a task type (e.g., classification, regression, learning-curve analysis, data-stream classification, survival analysis) and specific inputs and outputs expected for that type of task. For instance, for classification, the inputs would include a specific dataset and a reference to the labels (e.g., a target column in a tabular dataset). Additionally, one could define sensitive columns to be masked out or an evaluation measure that is of interest (although OpenML always computes many evaluation measures, so one can filter on them later). Second, to ensure reproducible evaluations, tasks include training and test splits such as hold-out or cross-validation.

#### Flows

Flows describe algorithms, scripts, tools, and anything else that can be used to train models and generate predictions. A flow description includes the name, description, hyperparameters of the algorithm or script parameters, and a list of dependencies. The goal of creating a flow is to allow an algorithm (configuration) to be understood and recreated faithfully. There are a few integrations with popular ML libraries, like scikit-learn,^12^ that allow the creation of flows automatically from algorithms defined by those libraries. In these cases, it is also possible to automatically recreate a model from a flow description, although reproducing custom flows is a challenge we are planning to address in the future.

#### Runs

When applying a flow to a task, i.e., training a model and making predictions for a specific dataset and data split, the resulting data are captured in a run. Run data include predictions of the model, the hyperparameter configuration used for training, and possibly also other information, such as measurements of wall clock or processor time, or locally computed evaluation measures. One of the core design elements of OpenML is that a run is always performed on the computer or the server of the user. The client downloads all important concepts (i.e., the dataset object, the task object, and data splits) to the local machine, initiates and trains a model, and makes predictions for the test instances. These predictions, together with information about the flow that produced them, are then uploaded back to the OpenML server. That allows for flexibility and scalability.

When a run is uploaded to the OpenML server, several performance measures will be calculated by the OpenML server based on the uploaded predictions. For example, it will automatically calculate the accuracy, per-class precision and recall, and f-measure for runs performed on classification tasks and the root-mean-square error for a run on a regression task. All of the run data, including its computed metadata, are also available through the API and may be used to, e.g., study the effect of hyperparameter tuning for an algorithm across different runs.^13^^,^^14^

#### Collections

Collections are sets of OpenML objects of the same type that belong together. They were originally introduced as benchmarking suites (for collections of tasks) and studies (for collections of runs),^8^ and those are currently also the only supported collections.

Collections provide an easy way for communities to share and reuse, e.g., benchmarks. Prominent examples include the benchmarking suites OpenML Curated Classification benchmark (OpenML-CC18^8^), OpenML Curated Tabular Regression benchmark (OpenML-CTR23^15^), the AutoML benchmarking suites,^16^ and Meta-Album.^9^ The OpenML-CC18 benchmarking suite, for example, is a collection of 72 different tasks that meet specific criteria, such as dataset size. For clarity, we refer to collections of tasks as benchmarking suites in the remainder of this paper, as it is the more commonly used name.

### Infrastructure

**Figure 2:**
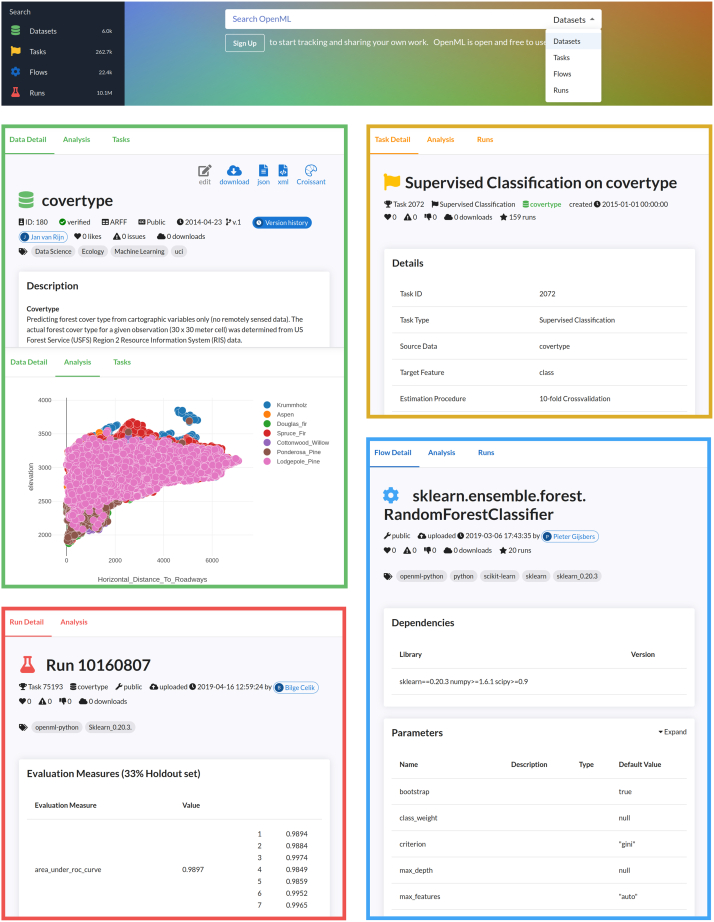
An overview of various pages of the OpenML website On top, the search bar of the landing page and the menu are shown, enabling users to explore the datasets and ML experiments. Next, a view is displayed of each of the four main concepts used in OpenML. Clockwise from top left: first, two pages of the CoverType dataset: one displaying dataset details and another showcasing a scatterplot of the class distribution plotted against two features, which is part of the analysis page. Second, a 10-fold cross-validation task for this dataset is depicted (top right), which can be combined with a random forest classifier from scikit-learn (the flow, bottom right), resulting in an area under the curve, f-measure, and other evaluation measures in the run (bottom left).

The OpenML website and libraries are the main user-facing components. From the website, the user can browse the datasets, tasks, and other concepts, along with their relations, as illustrated in Figure 2. From the overview pages, metadata are displayed and available in various standardized formats. The user is assisted in preliminary analyses by visualizing, among others, the feature statistics of datasets and the results of runs. These visualizations are also entirely open source and can be extended by the community.

To perform ML experiments, the user can interact with OpenML through software libraries to obtain the relevant resources, such as the dataset and the metadata for validation splits. The user then runs the experiment, and may report the results, in the form of a run, back to the OpenML platform. Software libraries are available in Python,^17^ R,^18^^,^^19^ and Java.^20^ The libraries allow the user to load, use, and publish datasets, tasks, flows, and runs with just a few lines of code. Independently developed packages can also be found for Julia, .NET, and Rust, but often offer limited functionality (e.g., only allowing to download datasets).

**Figure 3:**
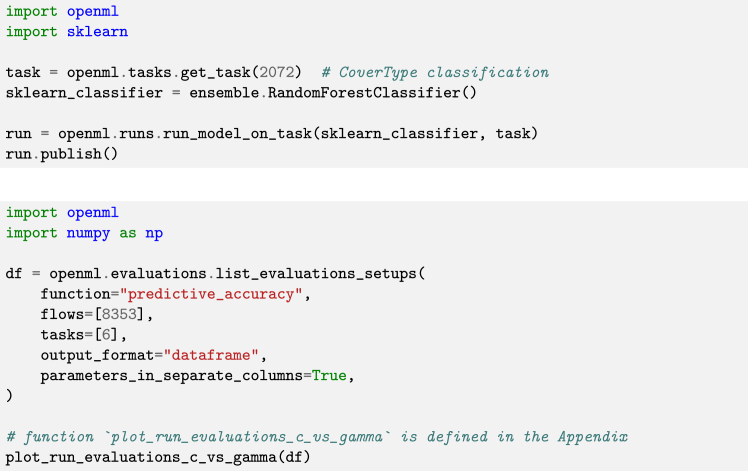
Example scripts using "openml-python" (version 0.15.1) Top: example code of the Python API using scikit-learn, applying a random forest classifier to an OpenML task and publishing the results on OpenML. Bottom: code to retrieve the results of all runs that apply flow 8353 (a scikit-learn pipeline containing a support vector machine) on task 6 (classification on the “letter” dataset). The visualization subroutine plot_run_evaluations_c_vs_gamma can be found in the supplemental information. Figure 4 shows the resulting plot.

**Figure 4:**
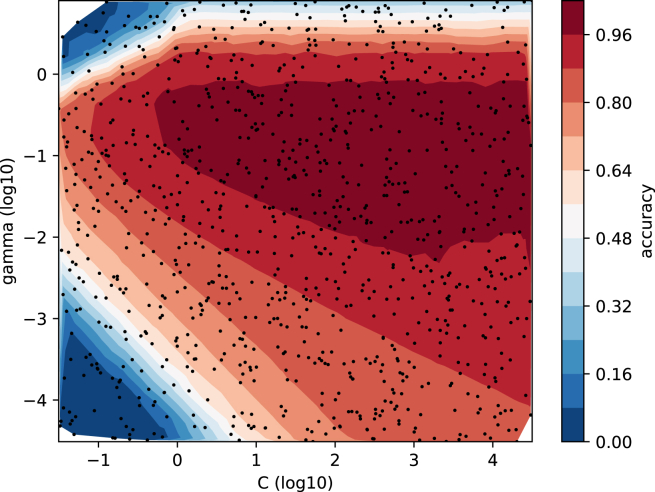
The support vector machine performance landscape generated by Listing 3 Using hundreds of evaluations downloaded from OpenML, represented by black dots, we visualize the effect of hyperparameters C (*x* axis) and gamma (*y* axis) on the model accuracy (hue). Figure adapted from Feurer et al.^17^

While any ML model may be used in experiments, OpenML integrates with popular ML frameworks to make automatic evaluation easy. In Python, models from scikit-learn,^12^ Tensorflow,^21^ and PyTorch^22^ can be used; in R, models from mlr3^23^ and its predecessor mlr^24^; and, in Java, WEKA.^25^ A model defined in any of these frameworks can be easily applied to any dataset or task from OpenML. Figure 3 (top) shows simple example code that evaluates an ML model from a supported library. Figure 3 (bottom) illustrates the advantages of having access to OpenML run results: without running any new experiments, we can, for instance, study the effect of hyperparameter configurations on model performance, visualized in Figure 4. Vice versa, frameworks such as scikit-learn^12^ (module: sklearn.datasets.fetch_openml) and PyTorch^22^ (module: torchrl.envs.OpenMLEnv) offer data loaders for OpenML to simplify dataset retrieval for users familiar with their respective code bases.

**Figure 5:**
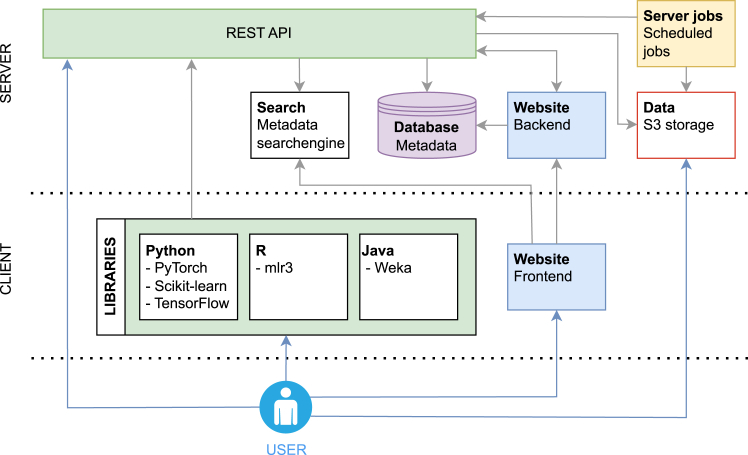
An overview of the OpenML components Every component of the OpenML ecosystem is depicted as a block. Arrows visualize communication, such as data retrieval or updates, whereby the arrow originates from the component that initiates the communication. Users can choose to interact via the website, via OpenML libraries in multiple programming languages, or interact with the REST API directly. The website includes a search engine and interacts with the back end for user authentication and actions such as uploading datasets and creating tasks. Metadata are stored in an SQL database, and files (e.g., datasets and models) are stored in an object store. Scheduled server jobs do various tasks such as analyzing datasets and evaluating runs.

Figure 5 shows the general system architecture of the various components of OpenML. Users typically interact via the website or client libraries, while, under the hood, multiple components make up the core of the OpenML ecosystem. The actual data reside on-premise in S3 buckets, the metadata in databases and in a search engine. Additionally, the REST API offers a uniform interface to access OpenML (meta)data and serves as a single control mechanism to ensure data quality. Finally, services periodically poll the metadata to perform tasks such as data conversion, meta-feature calculation, and evaluation of recently uploaded runs.

### Reproducibility

Reproducibility is a cornerstone of scientific progress but remains particularly challenging in empirical ML due to the complexity and variability of experimental setups.^2^ OpenML was designed with reproducibility in mind and aims to directly address these challenges by providing a collaborative platform that facilitates the sharing, organization, and reuse of ML experiments. Through its standardized APIs, OpenML enables researchers to upload datasets, flows, and runs in a structured and machine-readable format. OpenML aims to foster reproducibility in the following ways.

Open and FAIR principles: OpenML ensures that all data, tasks, flows, and runs are easily discoverable, have unique IDs, and are usable across different platforms, tools, and frameworks, complying with FAIR principles.^5^

Consistent APIs and convenient tooling: the APIs produce consistent metadata for every shared resource. This simplifies code, removes hurdles to adoption, and enables users to follow reproducible patterns in their code with less time investment or in-depth expertise.

Predefined data splits: OpenML provides predefined splits for tasks, such as k-fold cross-validation or hold-out validation sets, ensuring consistency across studies, making experiments comparable and eliminating ambiguities in experimental setups.

Experiment logging: using OpenML’s APIs, each run will be recorded with rich metadata, such as wall clock or processor time, and can easily be uploaded to OpenML.

Benchmarking suites: curated benchmark suites (i.e., collections of tasks and datasets) provide researchers with reproducible baselines for comparison, reducing the cherry-picking of benchmarks and allowing more standardized setups.

Together, these features lower the barriers to reproducibility in ML research, fostering more robust scientific inquiry and enabling faster progress through collaborative validation and reuse of prior work. However, it is important to note that, while OpenML includes many features designed to enhance reproducibility, it can by itself not guarantee full reproducibility.

Long-term reproducibility remains a significant challenge for the larger ML community. Precisely specifying code so that it remains runnable many years into the future is inherently difficult, as its dependencies may have updates with breaking changes or become unavailable altogether. Containerization (e.g., packaging the requisite Python, scikit-learn, and OpenML client versions in a Docker image) can mitigate these issues to a large extent. However, when containers require contact with the outside world (e.g., to download datasets), the static interface of the legacy container may become misaligned with the outside world as its servers move or receive updates to their interface.

We identify two possible solutions to this problem. One should either make the container self-contained (e.g., by also including all the data within) or you need to guarantee that the servers with which it communicates maintain full backward compatibility indefinitely. For a while, OpenML ensured this full backward compatibility as it reduces the burden on the researcher and allows for smaller research artifacts. However, in a rapidly evolving field such as ML, this scenario created an unsustainable burden on the project, particularly given its voluntary nature and limited resources.

Instead, users are encouraged to view OpenML as a significant step toward improved reproducibility. Runs can make some research artifacts, such as predictions and models, directly available. Moreover, their metadata, such as the flow, task, and dataset used in the experiment, also provide much (but not all) of the critical information required to reproduce the experiment even in the absence of a container. Last but not least, we would like to point out that, from a scientific view, exact reproducibility does not guarantee inferential reproducibility, the actual goal we should strive for.^26^

### Community

OpenML is an open-source, community-driven project. All our code is open source, including the platform itself, and we welcome new contributors. Our meetings and hackathons are open to all. Like many other open-source projects, a community of volunteers develops and maintains OpenML. Most of OpenML’s core contributors are PhD students and academics, often working in empirical ML, benchmarking, AutoML, and ML software. They volunteer their time, most commonly during working hours, as the platform aligns with their research interests. We have a merit-based governance structure for decision-making and community interaction. The community engagement is mainly handled through Slack and GitHub.

To foster a stronger community, we organize multiple in-person, week-long hackathons every year, where the community comes together to work on the OpenML platform and have discussions to shape OpenML’s roadmap. These hackathons have proved to be highly effective in making significant platform-related decisions, such as the development of OpenML-Python and the transition of OpenML’s standard dataset format to Parquet. Over the past 4 years, we have also organized smaller, targeted mini-hackathons focused on specific subcommunities, such as those working on engineering aspects or a particular API.

We are fortunate to be able to find institutions willing to provide a venue free of charge, often rooms in universities of our core contributors, and that many members of our community are able and willing to provide their own travel and accommodation funds, most commonly through their employers. This means that we can host events on a shoe-string budget or none at all, which allows us to have them more frequently. We also explicitly keep community building in mind during the hackathons, and, as such, we often organize at least one social event and plan for common lunches and dinners.

The majority of long-lasting active community participants (e.g., contributors) come from in-person contact, such as when we get the opportunity to speak at events and conferences, through the hackathons we organize, or through a place of work. However, there are also cases where people join our online meetings, contact us through e-mail, or immediately provide a code contribution through a pull request, as a point of first contact.

### Funding challenges

So far, OpenML’s funding has mostly been in kind, such as server and network infrastructure provided formerly by Leiden University and currently by the Eindhoven University of Technology, individual contributors being allowed to make contributions during their working hours and being provided with funding for attending workshops, or institutions offering to host our hackathons free of charge (for a more complete overview, see the acknowledgments section). While we are grateful for these contributions, they are often *ad hoc* and irregular, which makes developing and sustaining OpenML as a long-term service challenging.

At a bare minimum, OpenML requires funding for hardware, as servers need to be replaced periodically, and maintenance, as hardware components and software updates need to be installed. However, realistically, a project of this scale also needs funding for one or a few full-time developers who can ensure the software remains useful as research in ML changes, provide support to users, and manage the community.

Unfortunately, funding for open science remains limited. Most research funding primarily supports research personnel rather than physical infrastructure and often focuses on specific stand-alone outputs that do not align with strengthening existing initiatives. Finally, research software-engineering staff are often excluded from funding, which makes securing funding for these critical roles more difficult. While open-source projects like OpenML offer very rewarding work, offering long-term career prospects is a critical issue that needs to be addressed.

Thankfully, we do see improvement in this area, with better recognition for other academic contributions (e.g., the declaration on research assessment [DORA]), open science becoming increasingly valued through both dedicated funding calls, and mandating open-science practices for research grants. However, prospects for long-term research software engineers in general, and with long-term projects in particular, are not directly addressed by these developments and so remain an open challenge.

## Related work

Constructing new ML algorithms and benchmarks is a strong focus of ML research, while optimizing ML pipelines for given data and tasks is a key aspect in applied data science. Popular ML libraries like scikit-learn,^12^ mlr,^23^^,^^24^^,^^27^ PyTorch,^22^ Weka,^25^ or JAX^28^ support this through unified interfaces and design of modular and composable components. Alongside these libraries, a sophisticated tooling ecosystem has sprung up over the years to drive empirical research into ever-better ML algorithms.^29^ We introduce several prominent examples in the remaining text and summarize them in Table 1. The resulting collection of tools comprises dataset and experiment management, model sharing, and competitions, and established the rapid, empirical side of machine research we see today.

**Table 1:** A selection of popular, generalist tooling platforms used in ML and their features

Feature	UCI	Kaggle	HuggingFace	W&B	MLflow	Sacred	Hydra	DVC	Codabench	Dynabench	OpenML
Data and task hub	✓	✓	✓	×	×	×	×	×	×	×	✓
Model and results hub	×	✓	✓	✓	✓	×	×	✓	×	×	✓
Experiment management	×	×	×	✓	✓	✓	✓	✓	×	×	×
Community benchmarking	×	✓	✓	×	×	×	×	×	✓	✓	✓

From the outset, ML had a strong empirical component.^30^ Datasets, the primary input to ML algorithms, capture what we aim to predict and measure.^31^ Improving predictive performance on these datasets, conducting rigorous scientific benchmarking, and understanding performance differences—including the influence of hyperparameter effects and modeling components—are all common goals in ML research. Reproducibility (and replicability) are important traits of experimental research as they serve as a core validation mechanism (see Herrmann et al.^2^ for a detailed discussion on the state of ML research). Providing rich artifacts increases reproducibility^3^ and reduces the complexity of new comparisons,^7^^,^^32^ allowing a fast, efficient, and robust scientific process. In addition, it enables meta-analyses, as illustrated in Figure 4, which let us take a bird’s-eye view of a hypothesis across methods, experiments, or datasets. Despite rapid progress, ML research is still plagued with reproducibility issues due to bad sharing practices that confound and limit the impact of the field.^2^^,^^3^^,^^33^

### ML data-sharing standards

A key part of interconnected ecosystems is shared concepts and interfaces that enable different components to interact. One way to formalize such concepts is ontologies, and the OpenML community has actively worked on a shared vocabulary to systematically describe and catalog datasets, models, and results. The first effort was an ontology for describing ML experiments called Exposé,^34^ which was later harmonized with other ontologies into the W3C standard MLSchema^35^ and still forms the vocabulary with which OpenML concepts such as datasets, tasks, flows, and experiment runs are described.

Continued efforts toward a lingua franca of ML artifacts have resulted in platform-agnostic schemas for describing ML datasets, such as Croissant.^36^ Based on Schema.org, it is now shared across the data repositories Kaggle, HuggingFace, Dataverse, and OpenML (see next subsection for a description).

### Data hubs and task repositories

Before OpenML, there existed a patchwork of public ML dataset repositories, especially the UCI Repository^37^ and MLData^38^ (now defunct), while a host of more specialized collections, like KEEL,^39^ domain-specific repositories,^40^^,^^41^ and dataset lists on researchers’ private webpages, such as Luis Torgo’s regression suite,^42^ serve particular research communities. OpenML was the first platform that provided unified access to most of these datasets, with unified formats and APIs. This example was followed by other repositories, such as PMLB.^43^ Kaggle and Hugging Face later also started hosting datasets and have become important hubs for sharing datasets. These repositories are in many ways complementary with OpenML, e.g., they provide more free-form sharing of datasets, but do not store all datasets in standardized data formats to allow universal data loading and more extensive APIs, like OpenML does by design. Hugging Face now does offer data loaders for many datasets. In this space, OpenML remains the only platform that is fully open source (including its infrastructure) and non-commercial, which allows it to build bridges with these platforms that benefit all, as we discuss below. More recently, dataset search engines like Google Dataset Search and AWS Open Data Registry conveniently index datasets from all over the web. That said, they currently do not describe ML tasks in machine-readable formats, nor do they support the tracking of experimental results on these datasets or enable collaborative analyses like OpenML does.

### Experiment management

Tools like Weights & Biases,^44^ MLflow,^45^ Sacred,^46^ Hydra,^47^ DVC,^48^ and others enable researchers to track, version, and orchestrate ML pipelines and experiments. These frameworks provide reproducible workflow management but typically focus on computational aspects rather than facilitating scientific collaboration. While they shine at tracking experiment metadata and managing dependencies, they generally lack integrated facilities for open collaboration or sharing and discovering datasets, models, and results across the research community.

### Model and results hubs

On the model and evaluation side, platforms like HuggingFace and Kaggle have emerged as popular destinations for sharing pre-trained models and associated results. Management tools like Weights & Biases or MLflow also provide sharing capabilities for these artifacts, albeit more focused on internal use within one organization or business. While these services excel at their specific functions, they often operate in isolation rather than providing an integrated environment across communities and the complete ML research life cycle.

### Community benchmarking

General-purpose challenge platforms like Codabench,^49^ Kaggle, or Dynabench^50^ provide infrastructure for standardized algorithm evaluation. Specialized hosted benchmarks also exist, such as AlgoPerf,^51^^,^^52^ DataPerf,^53^ or Chatbot Arena,^54^ which cater to gradient-based optimization, data-centric, and LLM communities, respectively. While these platforms enable comparative evaluation, they typically focus on competition rather than collaboration. Although competition can be an effective motivation, it can also lead to obfuscating intermediate and negative results as well as incremental improvements.

In the landscape of ML tooling, OpenML provides an integrated platform that connects many aspects of ML research, from dataset sharing to experiment tracking to collaborative analysis. It has similarities with Kaggle or HuggingFace (Table 1) in this regard. Still, it differs from these other platforms with its particular emphasis on networked science principles, enabling organic collaboration while maintaining scientific rigor through standardized tasks and evaluations. The platform complements existing tools by providing APIs that allow integration with popular frameworks while adding collaborative capabilities that emerge from connecting researchers, datasets, and results into a unified system.

### Breaking the silos

The OpenML platform is designed to avoid becoming another isolated data silo by actively integrating with other platforms like Hugging Face and Kaggle to create an interconnected ecosystem. This is achieved through several initiatives.

(1) Development and adoption of community standards. We co-created the Croissant metadata format^36^ for datasets in collaboration with Kaggle, HuggingFace, and Google Dataset Search. This facilitates data interoperability and enables efficient data and model sharing between infrastructures.
(2) APIs. OpenML has extensive APIs in multiple languages (e.g., Python, R, Julia), which in turn allow integration into ML libraries (e.g., scikit-learn,^12^ PyTorch,^22^ TensorFlow,^21^ mlr3,^23^ and MLJ^55^).
(3) Direct integration with other platforms. OpenML and Kaggle collaborate to identify and link datasets hosted on both platforms, making it easier for users to navigate between related dataset pages. As such, Kaggle users can find benchmarks of those datasets on OpenML, and OpenML users can find notebooks analyzing these datasets on Kaggle.
(4) Pursuing complementary goals. OpenML aims to pioneer capabilities not yet available on other platforms. For instance, our APIs, uniform data formatting, and benchmarking tools facilitate the progressive automation of ML tasks and the creation of tabular and other foundational models.
(5) Focus on networked science principles. OpenML aims to create a “world-wide lab” to accelerate scientific research itself. It covers the entire ML life cycle, from datasets to models and evaluations, and allows people to organize all knowledge online. Much like Wikipedia, it allows people to build on top of each other’s work. While there are other tools to track ML experiments (e.g., MLFlow^45^), OpenML also allows people to build on top of the latest results of others and is unique in that respect.

On the other hand, it still lacks integration with other aspects of research, such as papers, code, and discussions. This is the focus of future work, and we plan to closely integrate with platforms that already offer those services, such as arXiv, PapersWithCode, MLFlow, and HuggingFace, to realize this. Some of this integration work may require significant changes to OpenML itself or for platforms to grow toward each other.

## Impact of OpenML

Over the years, OpenML has had a significant impact on the ML community, as shown by its widespread adoption, usage statistics, and numerous research papers citing OpenML-related works.^7^^,^^8^^,^^17^^,^^18^ Conferences like ECMLPKDD^56^ and the NeurIPS Datasets and Benchmark^57^ track also encourage authors to use OpenML, among a few other platforms, for open, reproducible research. To quantitatively assess this impact, we conducted a comprehensive literature analysis of papers that cite OpenML. In this section, we discuss the general trends we observed and highlight specific areas where OpenML has made an impact.

### Literature search

Using Google Scholar, we identified research papers citing the first core OpenML paper,^7^ the Python and R connectors,^17^^,^^18^ and the paper introducing benchmarking suites.^8^ In total, we collected 1,789 papers (published between 2014 and 2025), which we systematically analyzed to understand how OpenML has been used in research. The collected papers were distributed among all co-authors, who manually reviewed them and answered a questionnaire designed to capture key aspects of OpenML’s usage in the paper. Some papers were excluded from further analysis because they were not fully accessible, not available in English, or for other reasons documented. We have also excluded papers published in 2025 as the year is still in progress while writing the paper, to avoid skewed interpretations of trends. The questionnaire and selection process are documented in the supplemental information, and the final dataset as well as the analysis code are available online on GitHub.^58^ The final analysis is based on 1,528 papers. Our analysis focuses on the primary use cases of OpenML, including the use and publication of datasets, benchmarking suites, and experiments. We distinguish between papers that include at least one OpenML core contributor as co-author and those that do not. We also highlight papers that use OpenML in unique ways, were highly influential, or offer unique insights into the value OpenML can bring to ML research. In the following, we first provide general findings before surveying several fields of ML research that use OpenML in more detail.

### General findings

Table 2 presents the distribution of OpenML usage across various categories. Some papers use, e.g., both datasets and experiment data, so the sum of percentages exceeds 100%. Most papers that cite OpenML also interact with the technical platform in some way (instead of only discussing OpenML in text). We find that OpenML is predominantly used as a dataset repository, with 1,127 papers using OpenML datasets in experiments. Many of those include some evaluation of ML algorithms, but other use cases include evaluating meta-feature selection techniques,^59^ understanding performance evaluation measures,^60^ surrogate benchmarking,^61^ learning-curve analysis,^62^ and instance space analysis.^63^ Among the papers that only cite OpenML but do not actively use it, 90.4% reference the first OpenML paper,^7^ while the remaining 9.6% cite usage of one of the OpenML libraries.^8^^,^^17^^,^^18^

**Table 2:** Usage of OpenML in published research, based on papers that cite OpenML

Use cases	# of papers	% of papers	% co-authored by core members
Included in analysis	1,528	–	11.0
Only cite OpenML	319	20.9	7.2
Use datasets	1,127	73.8	11.0
Use benchmarking suites	194	12.7	27.8
Use experimental results	66	4.3	28.8
Upload datasets	23	1.5	69.6
Upload experimental data	27	1.8	74.1
Other interactions	88	5.8	28.4
Thesis publications	202	13.2	5.4

In addition to datasets, OpenML benchmarking suites have been used in 194 papers. These suites have been instrumental in tasks such as model comparison, hyperparameter optimization, meta-learning, and AutoML system evaluation.

**Figure 6:**
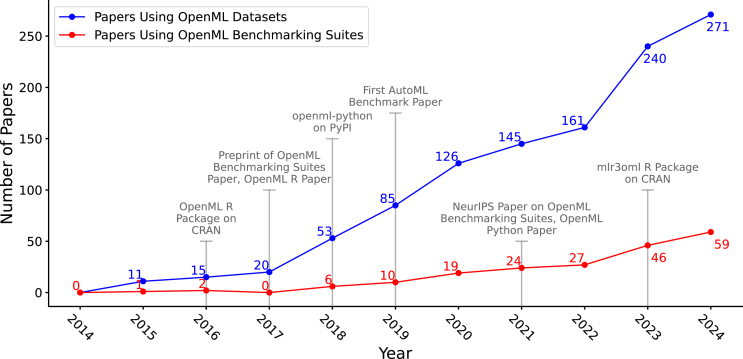
Number of new papers published each year using OpenML datasets and benchmark suites Gray markers denote milestones in the development of OpenML for context. Data from 2025 were excluded as the year was still in progress at the time of writing.

**Figure 7:**
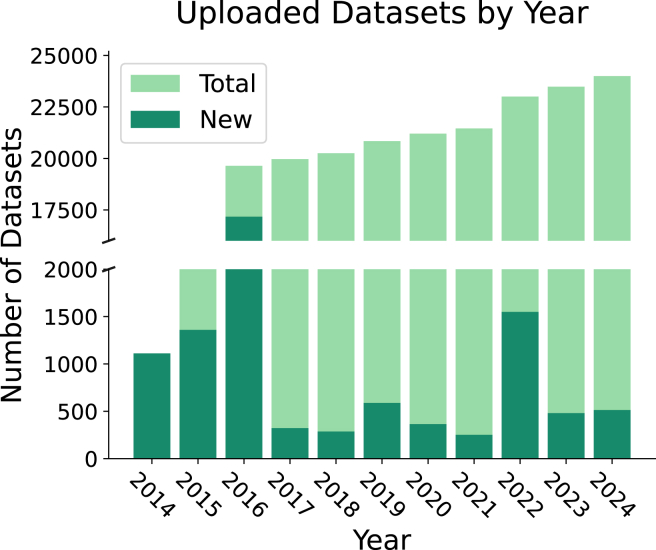
Number of datasets uploaded by year In 2014–2015, many datasets were being imported from older dataset repositories. In 2016, over 17,000 drug-discovery datasets were uploaded.^64^

**Figure 8:**
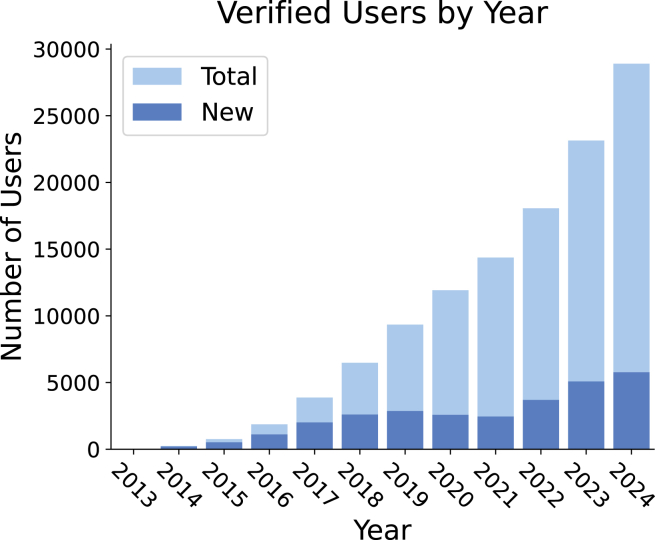
Number of email-verified OpenML users by year Note that registering is only required for uploading or getting an API key. Most users use OpenML without registering. For instance, the website has 300,000 unique yearly users.

Figure 6 shows the trends in using datasets and benchmarking suites over time. The consistent increase in both categories highlights the growing adoption of OpenML in ML research. This is further supported by usage statistics from the platform, which are shown in Figures 7 and 8.

OpenML also supports scientific progress by enabling researchers to build on previous work by reusing prior experiment data. A smaller subset of papers (4.3%) leverages OpenML’s experiment results to analyze algorithm performance or study hyperparameter tuning strategies. Approximately 3.3% of papers contribute to OpenML by uploading datasets or experimental results. This is a relatively small number, which may be due to several factors. First, the primary focus in ML research is the development of algorithms, which are often evaluated based on established datasets or benchmarks, so uploading new datasets to central repositories (instead of GitHub or a custom website) is less incentivized in this line of research. Second, creating new datasets is a labor-intensive task, which has only been getting more recognition in ML in recent years (e.g., due to dedicated publication venues such as the NeurIPS Datasets and Benchmark track). Moreover, while 73.8% of the papers use OpenML datasets, less than 2% of the articles have uploaded experimental results. Again, while researchers share their experimental results,^65^^,^^66^^,^^67^ most only include them in papers. There are currently few incentives for researchers to publish reproducible experimental results in data repositories. Additionally, OpenML requires experiments to be linked to both an existing task and a flow in order to upload them. This can be a substantial hurdle for users, which we aim to address in future work.

Finally, we find that 5.8% of papers make use of OpenML in a more advanced manner (beyond downloading data and running on curated benchmarking suites), such as creating benchmarking suites, integrating OpenML into novel ML frameworks, or using precomputed meta-features to construct a knowledge graph.^68^

Overall, many researchers are engaging in direct and indirect collaboration on the platform by using datasets, benchmarking suites, and experimental results published by other users in their own work. In the remainder of this section, we will highlight some of those individual works.

### Benchmarking and empirical analysis of ML algorithms

Having a vast base of experimental data containing ML experiments allows us to investigate the behavior of ML algorithms, under which circumstances they work, and why they work the way they do. In the experiment database, a preliminary version of OpenML, several of these patterns were already investigated,^69^ such as the performance of algorithms across datasets or the effect of dataset meta-features on hyperparameters.

OpenML and its software libraries have also been used to automatically create and store massive amounts of benchmark result data to enable further large-scale analysis. Purucker et al.^70^ use model predictions stored on OpenML to benchmark ensembles, requiring only a fraction of the cost of collecting the data without OpenML. Kühn et al.^71^ and Binder et al.^72^ collect massive amounts of performance data on hyperparameter configurations across datasets from OpenML. To illustrate, Binder et al.^72^ collected evaluations of seven different ML algorithms on up to 119 OpenML datasets from the OpenML-CC18 suite and the AutoML Benchmark (AMLB) for various hyperparameter configurations, resulting in more than 4 million model evaluations across many hyperparameter configurations over many different datasets (see below for uses of such data).

Furthermore, Codabench,^49^ a platform for ML competitions, treats benchmarking as a long-term competition and uses datasets from OpenML for many of their challenges.

OpenML has also been used to determine the effect of feature selection on classifier performance across datasets with varying sizes,^73^ as well as under which dataset sizes linear models compete with non-linear models,^74^ and even to propose a taxonomy of ML algorithms based on (dis)agreement in model predictions.^75^ In addition, the run data on OpenML can be analyzed to explore the modeling choices and iterative development of pipelines by its users.^76^ Lastly, the predictions available on OpenML can also be used to study the impact of calibration on different metrics, for example, for different class imbalances^77^ or to benchmark novel calibration methods,^78^ but the available predictions can also be used to better understand the behavior of metrics in ML.^60^ Recently, OpenML has had a positive impact on the trending research field “tabular deep learning,” where OpenML is used as a major source of test data.^65^^,^^79^^,^^80^^,^^81^

While the OpenML benchmarking suites^8^^,^^15^^,^^16^ received much attention, researchers are investigating them further. Cardoso et al.^82^ use item-response theory to shrink the OpenML-CC18 by 50%. McElfresh et al.^65^ manually define performance criteria to obtain a suite of challenging datasets. Curating benchmarking suites will remain relevant, as the research community has not yet settled on proper design techniques for benchmarking suites.^83^

### Meta-learning

The term meta-learning is slightly overloaded as it has been used by multiple communities. On one hand, meta-learning is used in the context of the algorithm-selection problem, where an ML model (the meta-model) is trained to predict which model would perform best on a given dataset, based on characteristics of the dataset.^11^^,^^84^ On the other hand, meta-learning is used as a method to transfer knowledge of a given (gradient-based) model across datasets.^85^^,^^86^ OpenML has proved very useful for both types of meta-learning.

For traditional algorithm selection, OpenML provides a large, diverse repository of datasets, their meta-features, and experimental results,^11^ and several meta-datasets have been developed to train meta-models. For example, the datasets on OpenML can be used to build data-preprocessing assistants.^87^ Notably, a meta-dataset from OpenML (OpenML-Weka-2017^20^) has been used in the open algorithm selection competition 2017.^88^ This dataset consists of 105 distinct datasets, 17 Weka algorithms, and 105 meta-features. To extend the density of datasets, Zabashta and Filchenkov^89^ propose to generate new datasets for meta-learning via active learning and demonstrate that this can result in better algorithm-selection performance. Carvalho et al.^90^ analyze the availability of meta-features across OpenML datasets and conclude that there is a discrepancy in the number of meta-features available per dataset. They extract a meta-dataset consisting of 459 datasets, 7 Weka algorithms, and 14 meta-features that can be used for meta-learning experiments. Olier et al.^64^ uploaded various quantitative structure-analysis relationship (QSAR) datasets to OpenML in various different representations, and they performed a meta-learning experiment to predict which representation and algorithm would perform best for a given dataset. This experiment was later extended to incorporate multi-task learning.^91^

While transfer learning for gradient-based models is typically performed on image-based tasks (due to the homogeneity of this type of tasks), datasets from OpenML have been used in the development of a meta-learning system that can be applied to tabular data.^92^^,^^93^^,^^94^ A particular complexity of working with tabular data is the heterogeneity of the datasets. Various tasks have different numbers of input variables and different numbers of classes, making it complex to utilize a single architecture across all tasks. The Chameleon algorithm utilizes a dataset encoder that represents datasets into a latent feature space such that the meta-learning method Reptile^95^ can be applied to it.

More recently, in the realm of foundation models, XTab^93^ pre-trains a transformer architecture across datasets from the AMLB,^16^ while TabDPT^94^ is a TabPFN-style model^79^ that is trained on datasets from OpenML instead of synthetic data.

### AutoML

AutoML research, which aims to construct optimal systems based on realistic evidence, is often driven by (automated) access to reliable empirical data. Thus, the AutoML community often interacted with OpenML and was among its first users. In 2015, Feurer et al.^96^ extensively used OpenML to develop Auto-sklearn, fetching 140 datasets for meta-learning and evaluation. As a matter of fact, the need for extensive metadata in the development of Auto-sklearn was the initial motivation for the development of the Python API. Moreover, with the introduction of the AMLB,^16^ data from OpenML became the *de facto* standard for benchmarking tabular AutoML systems.^81^^,^^97^^,^^98^^,^^99^^,^^100^^,^^101^ The AMLB consists of software that can automatically create the experiment environment, e.g., by downloading and installing AutoML software, and it combines this with OpenML benchmarking suites for automatic and reproducible data loading. The AMLB is also used in integration tests of industrial AutoML software.^98^

OpenML has also been used considerably in empirical research regarding hyperparameter optimization and the study of hyperparameter effects and importance. Van Rijn and Hutter^14^ use functional ANOVA across performance results obtained from all datasets in the OpenML100 benchmark suite to determine which hyperparameters of different learners are important and need to be tuned. Additionally, they use experimental data to infer good priors to sample from during hyperparameter optimization. Probst et al.^13^ determine which hyperparameters are important by defining a measure dubbed “tunability” and calculate it over a subset of the OpenML-100 benchmark. They also suggest a way to automatically construct search spaces based on the same data.

The above-mentioned data of Kühn et al.^71^ were in turn used by Perrone et al.^102^ to build surrogate models for benchmarking hyperparameter optimization algorithms. HPO-B,^103^ using the same data, and YAHPO Gym,^61^ using the data from Binder et al.,^72^ go even further and use large-scale results data from OpenML to build full benchmarking platforms for hyperparameter-optimization algorithms, releasing tabular and surrogate benchmarks for hyperparameter optimization.

In the realm of hyperparameter optimization, Feurer et al.^104^ explore finding well-performing ML pipelines on similar datasets using datasets from OpenML. Datasets from OpenML are also used to construct small sets of optimal hyperparameter configurations that can be searched sequentially and efficiently as a convenient alternative to more expensive and complex search algorithms.^66^^,^^96^^,^^105^ To avoid the need to evaluate multiple hyperparameter configurations altogether, Van Rijn et al.^106^ and Gijsbers et al.^107^ instead use data from OpenML to try to automatically find a single hyperparameter configuration that may be expressed as a function of dataset meta-features, providing a default configuration that adapts to dataset characteristics. Finally, the hyperparameter configurations found via meta-learning play an integral part in the AutoML systems Auto-sklearn,^96^^,^^104^ Auto-sklearn 2.0,^100^ and AutoGluon,^66^^,^^98^ the latter being the default ML toolkit inside Amazon, used by thousands of internal and external users.

### Education

OpenML’s easy access to toy and realistic data, and, in its combination with standard ML toolkits, convenient tooling has many benefits for education, as students can easily run their own experiments and deepen their knowledge about ML in a hands-on manner. Through the software packages provided, students can access thousands of datasets in a homogeneous way. This allows educators to let students experiment with ML much more easily and eliminate interruptions in the learning process. For instance, a search on coursehero.com yields hundreds of instances where OpenML is used in education, especially for lab work and assignments.

OpenML also allows “classroom challenges,” where students compete in building the best models, with a leaderboard on OpenML (available for any OpenML task), and afterward share the models so that other students can see the best solutions and learn from them. OpenML is also frequently used in online tutorials, such as those for scikit-learn and Fairlearn. Finally, we found almost 200 thesis publications where OpenML was referenced, usually because OpenML was used as part of their research. Note that these only include the theses that are indexed by Google Scholar, as in reality there might be many more theses that acknowledge the use of OpenML.

## Discussion

### Take-aways from building and maintaining OpenML

Looking back, there are several lessons we can draw from our work.

First, establishing a common vocabulary and standards to describe ML datasets, tasks, models, and results is crucial to facilitate a free exchange of resources and findings. This is never easy because scientific fields, tools, and platforms evolve quickly. We updated our schema three times in the last decade (with Exposé, ML-Schema, and Croissant). Bringing all major data hubs together to define a common standard and to keep the standard as simple as possible, as we did with Croissant, has proved helpful in gaining adoption and building bridges between platforms. Croissant only covers datasets, but initiatives are underway to extend it to tasks and model evaluations as well.

Second, providing open APIs and libraries in multiple languages has allowed users to easily use and share resources directly from their favorite tools with minimal code, without concern for the underlying storage mechanisms. For instance, when someone creates a dataset in a pandas dataframe, they can share it via the Python API, which stores it as Parquet files on the OpenML servers. Another scientist can import that dataset directly into PyTorch to train an ML model and then share the model and evaluations on OpenML. This also automatically links datasets to models and their evaluations and ensures rich metadata without additional work. Moreover, by using a language-agnostic REST API and language-specific APIs, OpenML can naturally adapt as new tools and languages become popular. This does require a continuous drive to develop new APIs and tool integrations and to make the infrastructure easily extensible and maintainable to stay abreast with the fast-moving field.

Third, reproducibility is *hard*. OpenML was designed with reproducibility in mind, providing features such as rich metadata, predefined tasks with data splits, and experiment logging via library integrations. Still, as ML tools evolve, old models (or flows) cannot always be loaded into new versions of these tools unless one containerizes every environment used, which does not scale. Moreover, the added complexity of registering tasks and models before experiments can be shared proves too much for many researchers. Logging experiments via callbacks (as in Weights&Biases or MLFLow) has proved much more practical but does not guarantee reproducibility, and no such tool has a popular hub for publicly sharing experiment logs. It is a difficult trade-off that probably requires new standards for sharing ML experiments and significant adoption of these standards in ML libraries.

Fourth, platforms such as OpenML require constant community engagement, welcoming new contributors and organizing open meetings and hackathons. During the COVID pandemic, the project felt a significant slowdown when such events could not be held and researchers had to focus on different tasks. Next to in-kind contributions, open-science platforms require funding for full-time developers to maintain the most critical software, provide user support, and manage the community. With most funding focused on research output, obtaining funding for such activities can be challenging. However, we do see a general trend toward more recognition of open science and the engineering effort that is required to support it. Therefore, we encourage anyone who finds OpenML helpful in their research to contribute, for example, by improving documentation, giving feedback, helping with code, or uploading ML artifacts.

Fifth, focus on what drives research forward. For instance, benchmarking suites such as OpenML-CC18, Meta-Album, and the AMLB have been instrumental in systematic model comparisons and driving research, especially in meta-learning and AutoML where a significant number of papers build on OpenML. Focusing on research creates incentives for scientists to invest effort in open science and helps them establish good careers. It also directly progresses scientific research. For instance, benchmarking suites increase reproducibility by standardizing evaluations, comparability by using the same suites across studies, and reliability by facilitating large-scale evaluations that are more general than hand-picking datasets.

Sixth, dataset curation is important. ML datasets may be incorrectly described (e.g., omitting crucial information about the variables or origin of the dataset). Tasks may be ill-defined (e.g., specifying the wrong target or input features), and run results can be flawed due to methodological errors such as test set leakage or improper seed tuning (e.g., selecting seeds to maximize accuracy). Using a curated data repository like OpenML helps avoid certain data-quality problems and ambiguities,^108^ but unreflected use can also undermine research quality.^109^ As open sharing of datasets is a foundational principle of open platforms, they typically lack an upfront formal review process, leaving potential for errors. However, (community-driven) quality control and the creation of curated task and run collections provide an effective remedy. These collections can serve as quality seals, enhancing trust for users who seek reliable results or demonstrate the trustworthiness of their own contributions.

Seventh, the datasets used by the ML community evolve over time. Changes may occur because previously unnoticed flaws are discovered or ethical considerations arise (e.g., Iris, Boston Housing). While we currently facilitate discussions of such issues via the openml-data repository (https://github.com/openml/openml-data), a more direct and integrated mechanism for flagging and discussing datasets would be beneficial (e.g., by treating each individual dataset as its own git repository and issue tracker).

Eighth, the ML landscape is constantly evolving. Methodological advancements have all but replaced hand-crafted feature extraction methods for vision, text, and speech with deep-learning methods that render tabular datasets with such extracted features obsolete.^108^ That said, tabular datasets are still highly relevant in many domains and play a new important role in the emergent field of tabular foundation models.^79^^,^^81^^,^^110^^,^^111^

Moreover, new methods make earlier problems (too) easy. For instance, computer vision classifiers can almost perfectly “solve” datasets such as CIFAR-10, CIFAR-100, and even Imagenet,^112^ and we see extremely fast saturation of new benchmarks for LLMs. The advent of foundation models that can memorize public datasets^113^ or that are pre-trained on public datasets will pose a significant issue for research, and this is a key open problem that requires our attention. More generally, reusing datasets over and over might lead to adaptive overfitting,^114^ even though we are not aware of any documented case of this. In all, creating and maintaining new, challenging benchmarks is a continuous effort and an important catalyst for innovation in ML.

Ninth, documenting the underlying data-generating process of experiments is important. For example, in our meta-analysis, we found that findings depend heavily on how tasks were selected, how flows and their hyperparameter configurations were determined, and the scientific motivation and practical context in which tasks, flows, and runs are created. This information exposes underlying assumptions, clarifies intended use cases, and helps gauge the external validity of any downstream analysis. If such information is not available (described in papers or available online), any analysis based on these results might be biased in various ways, thereby undermining the validity of scientific conclusions. Similar to point six, task and run collections offer a practical remedy by promoting transparent, standardized, and reproducible experimental settings. Moreover, systematic explorations of hyperparameters already exist on OpenML (e.g., see Kühn et al.^71^) and further enrich the evidence base for reliable meta-analysis.

Tenth, OpenML serves a surprisingly broad user community. While the primary focus has traditionally been to help scientists conduct empirical evaluations of ML algorithms, other important use cases emerged. For instance, scientists from other fields upload datasets to more quickly obtain good models of their data, while others might seek to identify effective starting models that perform well on similar, already-existing datasets. Other users share the results of benchmarking studies to be used by other researchers (e.g., as baselines). OpenML shows all run results alongside tasks to help with this, but we caution that these results also need to be interpreted with care due to optimization bias. An open challenge is to support the sharing of datasets with privacy concerns, as OpenML currently requires open licenses and ensures GDPR compliance. OpenML’s community is always eager to extend its functionalities to better address additional user scenarios, thus enhancing its utility and attracting a wider audience.

### Future

Looking forward, we aim to use these insights to further enhance OpenML’s functionality and empower the ML community. First, we aim to simplify the process of conducting reproducible yet more flexible research, enabling researchers to share and compare their findings easily. This involves streamlining the sharing of datasets, models, and benchmarks, making it easier for users to contribute to the platform and access a broader range of resources. More concretely, this encompasses several aspects, including better documentation support (e.g., datasheets^115^ and model cards^116^); increasing support for datasets of different modalities; code integrations such as callbacks in popular ML libraries, especially facilitating the integration of deep learning libraries to support reproducible benchmarks; and developing more collaboration tools (e.g., integration with Jupyter Notebooks and HPC resources). Another primary goal is to integrate even more with other platforms. This interoperability will be facilitated by adopting community standards such as Croissant, and we are already working with our Croissant partners to extend it toward ML tasks and benchmarks. Moreover, we also aspire to integrate with community platforms that offer additional resources such as papers, code, notebooks, experiment tracking, and discussions (including arXiv, PapersWithCode, MLFlow, Kaggle, and Hugging Face). We aim to avoid reinventing the wheel and instead create a more interconnected ecosystem and empower existing communities. All in all, we hope to realize the vision of a truly networked science environment for OpenML, especially by integrating OpenML as closely as possible with other dedicated platforms and research infrastructure.

## Resource availability

### Lead contact

Requests for further information and resources should be directed to and will be fulfilled by the lead contact, Joaquin Vanschoren (j.vanschoren@tue.nl).

### Materials availability

The source code of all pieces of software required to host the repository, of all clients, and also of several studies is available under open-source licenses at https://github.com/openml. OpenML, and the datasets, tasks, flows, runs, and collections are available at https://openml.org itself. Finally, the new material is available on GitHub as described in the next subsection.

### Data and code availability

• The final survey data, as well as all original code, are available at https://github.com/openml/OpenML-Paper-Impact-Analysis at https://doi.org/10.5281/zenodo.15464166 and are publicly available as of the date of publication.
• Any additional information required to reanalyze the data reported in this paper is available from the lead contact upon request.

## Acknowledgments

Throughout the lifetime of the project, spanning more than a decade, OpenML has received many contributions from volunteers. These wonderful people contributed their valuable free time, or had their own funding or employment, and took some of that time to contribute to OpenML when their work goals aligned with those of OpenML. We acknowledge and thank those contributors and the funds that made their contributions possible. Unfortunately, we cannot provide an exhaustive list here and instead defer to the acknowledgments in the research papers they produced. Special thanks go out to past core contributors who did substantial work for OpenML but did not co-author this paper: Heidi Seibold, Arlind Kadra, Neeratyoy Mallik, and Eddie Bergman. Heidi Seibold served on the steering committee of the Open Machine Learning Foundation (2018–2021). Arlind Kadra supported the Java API development (2017–2018) as well as the Python API (2018–2020). Neeratyoy Mallik supported the Python API (2019–2021). Eddie Bergman supported the Python API (2024–2025).

J.V., J.N.v.R., and B.B. have been part of OpenML from the beginning and led its initial design. In 2018, OpenML created the Open Machine Learning Foundation and a steering committee with B.B., G.C., M.F., Heidi Seibold, J.N.v.R., and Heidi Seibold resigned and P.G. joined in 2021.

L.P. acknowledges funding by the 10.13039/501100001659Deutsche Forschungsgemeinschaft (DFG, German Research Foundation) – project ID 499552394 – SFB 1597. M.W. acknowledges funding by the 10.13039/501100000780European Union (ERC, ixAutoML, grant no. 101041029). L.N. and S.F. were supported by Mathematische Forschungsdateninitiative (MaRDI), funded by the 10.13039/501100001659Deutsche Forschungsgemeinschaft (DFG), project number 460135501, NFDI 29/1. P.G., J.V., J.v.d.V., and T.D. acknowledge funding by EU’s 10.13039/100018693Horizon Europe research and innovation program under grant agreement no. 101070000 (AI4EUROPE). P.G., J.V., J.v.d.V., T.D., and J.N.v.R. acknowledge funding from the 10.13039/501100007601Horizon 2020 program under grant no. 952215 (TAILOR). S.M., J.N.v.R., and J.V. acknowledge funding from the Dutch national science fund (NWO) under grants 612.001.206 (Massively Collaborative Machine Learning) and OSF23.2.109 (Automated Machine Learning for All). J.V. acknowledges funding from an Amazon Researcher award.

Besides indirect funding through grants that have allowed contributors to contribute as part of their work, we also acknowledge the support that has been granted directly to OpenML in its lifetime, both monetary and in kind. We especially thank KU Leuven, Leiden University, and Eindhoven University of Technology for hosting the OpenML server infrastructure and many project events. We thank SURF, Microsoft Azure, and Amazon AWS for compute and hosting credits. Finally, we would like to thank the many institutes that supported OpenML hackathons, including the Dagstuhl Leibniz Center for Informatics, the Lorentz Center for Scientific Workshop, the Jheronimus Academy of Data Science, Probabl.ai, the Porto Business School, the 10.13039/501100007821University of Tartu, 10.13039/501100005722LMU Munich, and INRIA Paris.

Finally, we would like to thank Research Data Netherlands for awarding OpenML the Dutch Data Prize in 2016.

## Author contributions

Author names are listed alphabetically, and their order does not reflect the effort of their contributions to this paper or OpenML as a software project. We made that choice because of the size of the project, its long history, and the large body of authors. We describe the author contributions first from the point of view of contributions to the platform and community and then in terms of contributions to the paper.

The OpenML front end, back end, and API have been actively developed by J.V. and J.N.v.R. since OpenML’s inception in 2012, with J.N.v.R. leading API and back-end development while J.V. led front-end development and project management. S.R. and P.S. contributed to both front end and back end since 2019, P.G. since 2021, and T.D. since 2023. L.N. joined in 2024 and contributed to the front-end development. J.V. and J.N.v.R. initially set up the OpenML infrastructure, joined by P.S. in 2020, and J.v.d.V. took over in 2022. The Java API development was led by J.N.v.R. since 2013, M.W. (2020–), and J.v.d.V. (2024–), while the R API was originally initiated by B.B. in 2013, with G.C. taking primary development responsibilities since 2015 and S.F. joining in 2022 to develop a follow-up package. Development of the Python API was spearheaded by M.F. since 2014, joined by A.C.M. (2016–2019), J.N.v.R. and J.V. (2016–2020), P.G. (2018–). The OpenML-Python deep-learning extensions were developed by P.S. (2020–2024) and T.D. and S.M. (2024–). S.R. (2019–2021) contributed to the back end, Python API, and the OpenML website. Community and funding efforts were initially led by J.V. and B.B. (2012–), joined by P.G. (2021–). L.O. first joined in 2023 and contributed to the shared data format Croissant.

All authors contributed significantly to the writing of this paper, and all were involved in editing and extensive discussions on the scope of the paper. J.V. led the writing of the introduction, takeaways, and future work. M.F. and L.O. led the discussion of related work. Main authors for the “Building OpenML” section were, in order of subsections, P.G., J.v.d.V., M.W., P.S., P.S. and P.G., and J.V. The impact analysis was led by T.D. and J.N.v.R., with T.D. playing a pivotal role in distributing and compiling literature reviews and analyzing results. All authors contributed to reviewing papers for the impact analysis and the writing of that section.

## Declaration of interests

The authors declare no competing interests.

## Declaration of generative AI and AI-assisted technologies in the writing process

During the preparation of this work, the authors used ChatGPT and Grammarly to improve the writing style, grammar, and readability. After using these tools/services, the authors reviewed and edited the content and take full responsibility for the content of the published article.

## Supplemental information

Document S1. Appendix A and Appendix B

Document S2. Article plus supplemental information
