# Supplementary material for: OpenML: Insights from 10 years and more than a thousand papers
Source: Patterns (N Y). 2025 Jul 3;6(7):101317. doi: 10.1016/j.patter.2025.101317 (PMC12416095; doi:10.1016/j.patter.2025.101317)
Supplement: Document S1. Appendix A and Appendix B [file mmc1.pdf]

Patterns, Volume 6

Supplemental information

OpenML: Insights from 10 years and more than a thousand papers

Bernd Bischl, Giuseppe Casalicchio, Taniya Das, Matthias Feurer, Sebastian Fischer, Pieter Gijsbers, Subhaditya Mukherjee, Andreas C. Müller, László Németh, Luis Oala, Lennart Purucker, Sahithya Ravi, Jan N. van Rijn, Prabhant Singh, Joaquin Vanschoren, Jos van der Velde, and Marcel Wever

Supplementary Information

Appendix A: Reproducing Figures

The code in Listing S1 is called from Listing 1 to generate the plot in Figure 4.

```
import matplotlib.pyplot as plt
import numpy as np

def plot_run_evaluations_c_vs_gamma(df):
    hyperparameters = ["sklearn.svm.classes.SVC(16)_C", "sklearn.svm.classes.SVC(16)_gamma"]
    df[hyperparameters] = df[hyperparameters].astype(float).apply(np.log10)

    fig, ax = plt.subplots()

    C = df["sklearn.svm.classes.SVC(16)_C"]
    gamma = df["sklearn.svm.classes.SVC(16)_gamma"]
    score = df["value"]

    ax.plot(C, gamma, "ko", ms=1)
    cntr = ax.tricontourf(C, gamma, score, levels=12, cmap="RdBu_r")
    fig.colorbar(cntr, ax=ax, label="accuracy")
    ax.set(
        xlim=(min(C), max(C)),
        ylim=(min(gamma), max(gamma)),
        xlabel="C (log10)",
        ylabel="gamma (log10)",
    )
```

Listing S1. Plotting the results of various Runs, as used in Listing 1

Appendix B: Impact Analysis Methodology

For our literature analysis in Section 4, co-authors reviewed the papers collaboratively (at least 100 each), and were asked to answer to following questions for each paper:

- What is the paper title?
- What is the year of publication?
- Is the paper available?
- Is the paper in English?
- Is an OpenML core contributor co-author? If so, who?
- Does the paper use datasets from OpenML?
- Does the paper use benchmarking suites? If so, which?
- Does the paper use experiment data from OpenML (e.g., *runs*)? If yes, how?
- Does the paper upload datasets to OpenML? If yes, which?
- Does the paper upload experiment data to OpenML? If yes, what type of experiment data?
- Does the paper interact with OpenML in any other way? How?
- Should the paper be considered to be highlighted? If so, why?
- Is the paper a thesis?
- (optional) Provide a short description or remark.

The first questions are mainly to ensure the answers correspond to the expected paper and serve as a sanity check. In rare cases, citing OpenML only happens in some iterations of the paper, in which case answers are expected to be given with respect to that particular version of the paper. After conducting an initial analysis and sanity check, we filtered the papers based on specific criteria. The following exclusions were made:

- Total papers initially identified: 1786
- Papers not fully available: 184
- Papers not in English and without available translations: 72; 22 papers were both unavailable and not in English.
- Papers published after 2025: 24; We exclude papers published in 2025 as the year is still in progress, to avoid skewed interpretations of trends.
- Final papers analyzed: 1528
